# Microbiological Changes during Long-Storage of Beef Meat under Different Temperature and Vacuum-Packaging Conditions

**DOI:** 10.3390/foods12040694

**Published:** 2023-02-06

**Authors:** Pablo Rovira, Giannina Brugnini, Jesica Rodriguez, María C. Cabrera, Ali Saadoun, Guillermo de Souza, Santiago Luzardo, Caterina Rufo

**Affiliations:** 1 Sistema Ganadero Extensivo y Arroz-Ganadería, Instituto Nacional de Investigación Agropecuaria (INIA), Ruta 8 km 281, Treinta y Tres 33000, Uruguay; 2 Instituto Polo Tecnológico de Pando, Facultad de Química, Universidad de la República, By Pass de Pando y Ruta 8, Pando 91000, Uruguay; 3 Facultad de Agronomía Udelar, Avenida Garzón 861, Montevideo 12900, Uruguay; 4 Facultad de Ciencias, Udelar, Calle Iguá 4225, Montevideo 11400, Uruguay; 5 Sistema Ganadero Extensivo y Agroalimentos, Instituto Nacional de Investigación Agropecuaria (INIA), Estación Experimental INIA Tacuarembó, Ruta 5 km 386, Tacuarembó 45000, Uruguay

**Keywords:** meat shelf-life, chilled and frozen, microbiome, type of packaging

## Abstract

We evaluated a combination of two temperatures and two packaging materials for long-term storage of vacuum-packaged (VP) beef striploins. Microbial populations and microbiome composition were monitored during refrigerated storage (120 days between 0–1.5 °C) and refrigerated-then-frozen storage (28 days between 0–1.5 °C then 92 days at −20 °C) under low-O_2_ permeability VP and high-O_2_ permeability VP with an antimicrobial (VPAM). *Pseudomonas* (PSE) and *Enterobacteriaceae* (EB) counts in VPAM samples were significantly higher (*p* < 0.05) than in VP samples at 28, 45, 90, and 120 days of storage. Microbiome data showed that bacteria of the genera *Serratia* and *Brochothrix* were more abundant in VPAM samples at 120 days, while lactic acid bacteria (LAB) dominated in VP samples. Frozen temperatures inhibited microbial growth and maintained a relatively stable microbiome. Refrigerated and frozen VPAM samples showed the greatest difference in the predicted metabolic functions at the end of storage driven by the microbiome composition, dominated by PSE and LAB, respectively. Although no signs of visible meat deterioration were observed in any sample, this study suggests that VP meat refrigerated and then frozen achieved better microbiological indicators at the end of the storage period.

## 1. Introduction

With the globalization of the food markets, the export of meat is an important component of the Uruguayan economy. In 2020, Uruguay exported almost 80% of its total beef production to more than 50 countries [1]. One of the challenges currently facing the Uruguayan meat industry is extending the shelf-life of meat products [2]. A longer shelf-life can make Uruguayan exports more competitive by allowing products to retain their desired quality and appearance for longer, maximizing opportunities in distant markets and providing beef processors with more time flexibility. For example, Uruguayan meat sales to China reached 61% of the total meat exports in 2021 [3]. Completing and shipping a container overseas with protocolized high-quality beef meat from Uruguay to Asia takes several weeks. Moreover, in-store display and the time the meat product is kept in the consumer’s refrigerator extend the period in which meat should sustain its quality to fulfill the customer’s requirements at the time of consumption. Traditionally, informal evidence from international traders suggests a meat shelf life of around 100 days when vacuum packaged (VP) and stored at −1 °C [4]. 

During processing, transportation and storage, spoilage is a microbial process that happens and can render meat undesirable or unacceptable for human consumption due to changes in appearance and sensory characteristics [5,6,7]. Storage conditions, such as VP or modified-atmosphere packaging (MAP), chilled temperatures, and antimicrobial compounds have been used to inhibit or retard microbial growth in meat [8,9,10,11,12,13]. While the shelf-life of meat packaged in high-O_2_ MAP is approximately one week, the shelf life of VP meat is around three to 12 weeks when stored at 0 °C [14]. According to Liang et al. [15], the shelf-life of lamb meat in MAP conditions could be extended to over 70 days when stored between −4 °C and −9 °C. However, although lower temperatures increase shelf-life, sub-freezing or freezing conditions may deteriorate meat quality attributes after some time [16,17]. Research on active antimicrobial packaging, such as bacteriocin-activated plastic films or nisin-EDTA solution, have shown that spoilage associated microbial populations can be effectively inhibited in VP meat [18,19]. Recently, novel metallic-based antimicrobials have emerged to control bacterial growth in food products through different modes of action [20]. In the present study, we used a silver-based antimicrobial in the packaging material for which it has shown promising results to control bacterial growth in chicken breast fillets [21].

Because storage temperature and packaging material are effective ways to control microbial spoilage, the combination of those alternatives appears promising for the extension of the microbiological shelf life of VP meat [22]. However, this combined effect for prolonged periods of storage is unknown, as most studies have evaluated one single factor or were performed over a few weeks [18,19]. The microbial profile has been barely investigated in chilled-then-frozen meat, and it is particularly poor if we refer to long-term frozen storage following a shorter period of chilled storage [23,24]. In addition, the efficacy of storage conditions and packaging technologies in reducing or inhibiting microbial growth has been primarily studied using traditional culture-based methods [13]. While this approach is useful in understanding the change in the number of organisms belonging to specific groups of interest (i.e., *Pseudomonas* spp., lactic acid bacteria, *Enterobacteriaceae*, *Brochothrix thermosphacta*), culturing approaches do not capture the full diversity of the bacterial community (microbiome), which can promote or restrict the growth of the organisms of interest [25]. The development of culture-independent molecular methods, such as 16S rRNA gene sequencing, provides insights into microbial ecology, allowing the exploration of the full diversity, composition, and predicted functionality of meat microbiomes [25,26].

In this context, the aim of this work was to evaluate bacterial growth and microbial diversity of beef under different vacuum-packaging conditions and temperatures during long-term storage. We used culture-based techniques and 16S rRNA gene sequencing to evaluate the effect of treatments on the development and succession of specific spoilage organisms, the microbiome composition, and the predicted metabolic functions of the bacterial communities during storage. The hypothesis was that a combination of refrigeration and freezing temperatures would be more efficient to control microbial growth during the storage of vacuum-packaged meat instead of continuous refrigeration. Additionally, the addition of an active antimicrobial could control microbial growth in vacuum-packaged meat regardless of the oxygen permeability of the wrapping film.

## 2. Materials and Methods

### 2.1. Carcass Sampling and Experimental Treatments

Forty left striploins (*Longissimus lumborum* muscle) were collected from carcasses of steers fattened on high-concentrate diets intended for the EU 481 quota. The average (± SEM) hot carcass weight was 265.9 ± 3.2 kg. Slaughter took place in a commercial meat processing facility. Carcasses were graded after slaughter using the Uruguayan grading system established by the National Meat Institute (INAC) [27], and conformation, degree of finishing, and dentition data were recorded (Table 1). Different muscling grades were based on visual assessment of muscle mass development and were identified by the letters: I-N-A-C-U-R, from very muscular development to thinly muscled. The degree of finishing was evaluated by observing the amount and distribution of subcutaneous fat from lack of fat cover (0) to excessive fat cover (4).

**Figure 1:**
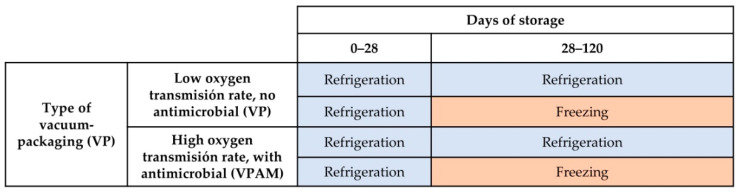
Treatments and experimental design.

Experimental treatments stem from the combination of two packaging types (vacuum packaging vs. vacuum packaging with an antimicrobial agent) and two storage conditions of meat (chilled for 120 days between 0 and 1.5 °C vs. chilled for 28 days and then frozen at −20 °C for 92 days), where 10 striploins corresponded to each treatment (n = 10) (Figure 1).

The following packaging options were evaluated: (1) vacuum packaging (VP) using a Supervac GK 842B (Supervac^®^ GmbH, Mödling, Austria) and a barrier bag (50 μm thickness; maximum oxygen transmission rate [OTR] of 27 cm^3^/m^2^/24 h at 22–24 °C and 0% RH and moisture vapor transmission rate [MVTR] of 5 g/m^2^/24 h at 38 °C and 90% RH; Cryovac^®^ Sealed Air Corp., BB 2620, Jaguariúna, Sao Paulo, Brazil); (2) vacuum packaging with an antimicrobial agent (VPAM) using a Multivac P605 (Multivac Inc., São Paulo, Brazil) and a polyamide bag (50 μm thickness; oxygen transmission rate [OTR] of 350 cm^3^/25 μm/m^2^/24 h at 23 °C and 85% RH and moisture vapor transmission rate [MVTR] of 58 g/25 μm/ m^2^/24 h at 23 °C and 85% RH; M&Q Packaging^®^, BioPlastic 11, Limerick, Ireland) with an antimicrobial component based on silver ion technology (Biomaster^®^, Addmaster Ltd., Stafford, United Kingdom) incorporated into the bag by extrusion.

Striploins from the left “pistola” cut (prepared from the hindquarter by removing the thin flank, lateral portion ribs, and a portion of the navel end brisket) of each carcass were fabricated after 48 h of slaughter by cutting from the 10th rib to the lumbar-sacral junction and then were trimmed to approximately 1 cm of external fat thickness. Subsequently, each strip loin was cut into five pieces of 6-cm-thick corresponding to one of the five time periods in which measurements were performed: two days post-mortem (day 0) and 28, 45, 90, and 120 days of storage. The cranial piece of each strip loin was used for the two-day post-mortem evaluation (day zero), and then each piece was randomly assigned to one of the four storage periods (28, 45, 90, and 120 days) within each strip loin. Once each storage period was completed, a 1.5 cm thick steak was cut from the 6 cm-thick strip loin pieces from the cranial end for microbiological counts and microbiome characterization. Knives and saw blades were sanitized during sample processing to avoid cross-contamination.

### 2.2. Microbiological Determinations and DNA Extraction

Meat samples were homogenized in sterile Phosphate Buffered Saline (PBS), pH 7.4 (Sigma) with a ratio of mass/volume (5:1) using a Seward Stomacher^®^ 400. The homogenates were used for both bacterial enumeration and DNA extraction for high-throughput sequencing.

For microbiological analysis, 10-fold dilution series were carried out. For total viable counts (TVC), diluted samples were cultured on duplicate plates of Plate Count Agar (Oxoid Ltd., Basingstoke, Hampshire, United Kingdom) and incubated at 37 °C for 48 h. *Enterobacteriaceae bacterium* (EB) numbers were determined on duplicate plates of Violet Red Bile Glucose Agar (Oxoid Ltd.) and incubated at 37 °C for 24 h. Lactic acid bacteria (LAB) numbers were determined on duplicate plates of De Man, Rogosa, Sharpe Agar (Merck, Darmstadt, Germany) and incubated anaerobically at 30 °C for 72 h. *Pseudomonas* spp. was determined on duplicate plates of Pseudomonas Agar Base (Oxoid Ltd.) supplemented with CFC selective agar supplement (Oxoid Ltd.) incubated at 30 °C for 48 h. Results were expressed as log (CFU/g) of sample. When no bacteria were detected, a log value of half of the detection limit was used for the calculation of the mean number. Bacterial log counts were analyzed using a one-way ANOVA to compare temporal and treatment changes, and means were compared using Fisher’s LSD post hoc test using InfoStat software. All statistical results were accepted as significant at *p* < 0.05.

For DNA extraction, 40 mL of the homogenate were centrifuged at 10,000× *g* for 10 min at 4 °C to pellet intact cells. The resulting supernatant was discarded, and pellets were stored at −80 °C until being processed for DNA isolation. For DNA extraction and further sequencing, samples were processed in pools of two. For this, two cell pellets were combined, and total DNA extraction was performed with DNeasy Power Fecal Microbial Kit (Qiagen), according to the manufacturer’s instructions. DNA was eluted in 50 μL of buffer solution and filtered through the spin column twice to optimize yield. DNA purity and quantity were measured by the Nanodrop ND-1000 Spectrophotometer (Thermo Fisher Scientific, Watertown, MA, USA). Purified DNA was stored at −20 °C until sequenced. Four pooled samples per treatment, and time points were selected according to the amount of DNA (more than 50 ng/uL) and quality (A260/A280 between 1.8–1.9). 

### 2.3. 16S rRNA Amplicon Sequencing and Microbiome Data Analysis

Microbial community structure was investigated by 16S rRNA amplicon sequencing of total extracted DNA. Samples were sequenced on an Illumina MiSeq 300 bp paired-end (PE) at Macrogen Inc. (Seoul, Republic of Korea) using primers targeting the V3-V4 variable region (341F 5′-CCTACGGGNGGCWGCAG-3′ and 805R 5′-GACTACHVGGGTATCTAATCC-3′) on the 16S rRNA gene. 

Raw sequences were received as PE reads in Fastq format and were processed with DADA2 [28] to quality filter, trim, denoise, merge the PE reads, and infer amplicon sequence variants (ASV). After the removal of chimeric sequences, the obtained ASVs were mapped to the SILVA database v138.1 [29] for taxonomic classification. The DECIPHER package [30] was used for the alignment of multiple ASV sequences, and a phylogenetic tree was built using the phanghorn package [31]. The phyloseq package [32] was used to combine sample metadata, ASV table, phylogeny, and taxonomic assignment objects into a single phyloseq object. Calculations of alpha diversity (Chao1 and evenness) and sample similarity based on weighted and unweighted UniFrac distances (beta diversity) were performed with the phyloseq package [32]. The Venn diagram illustrating ASV overlapping between groups was generated using the VennDiagram package. The potential function of microbiomes at the end of storage was predicted by the phylogenetic investigation of communities by reconstruction of unobserved states (PICRUSt2) [33]. Predicted metagenome functions were performed at level 2 and 3 KEGG pathways.

### 2.4. Statistical Analysis of Microbiome Results

Statistical analysis of data was carried out using R statistical software (version 4.0.5) with a *p* < 0.05 significance level to evaluate differences in the microbiome composition among treatments and sampling days. The alpha diversity index Chao1 was statistically tested using the non-parametric Kruskal-Wallis test followed by Wilcoxon rank sum tests to perform pairwise comparisons. Statistical analysis of microbiome beta diversity was performed using permutational multivariate analysis of variance (PERMANOVA) using the adonis function in the R package vegan. Analysis of differentially abundant taxa among the treatments at the genus level was determined using the package DESeq2 based on the negative binomial distribution method [34]. Statistical differences in microbiome functionality (PICRUSt2) between treatments were determined using the two-sided Welch’s test, and Benjamini-Hochberg FDR was used to correct for multiple tests in the Statistical Analysis of Metagenomic Profiles (STAMP) software [35].

## 3. Results

### 3.1. Bacterial Growth in Vacuum-Packaged Chilled and Frozen Beef

**Figure 2:**
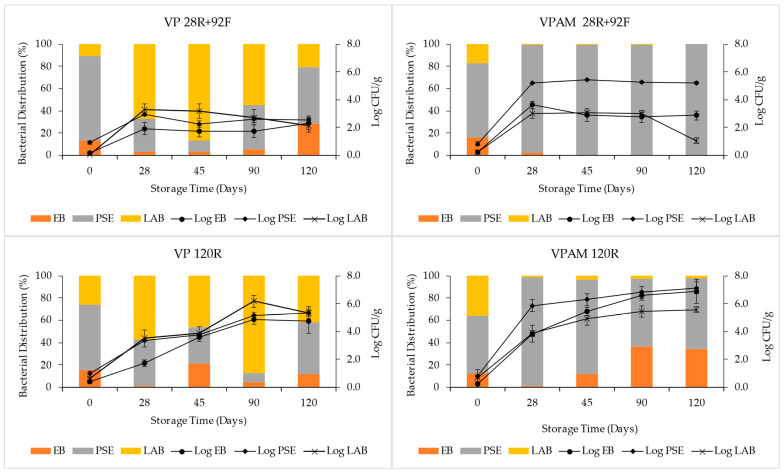
Growth curve and distribution of *Enterobacteriaceae* (EB) *Pseudomonads* sp. (PSE), and lactic acid bacteria (LAB) over time in beef treated with different packaging and refrigeration systems stored for 120 days. Treatments: vacuum packaging for 120 days of refrigeration (VP 120R), vacuum packaging for 28 days of refrigeration + 92 days frozen (VP 28R+92F), vacuum packaging with 120 days antimicrobial refrigeration (VPAM 120R), vacuum packaging with antimicrobial for 28 days refrigeration + 92 days frozen (VPAM 28R+92F). log CFU/g, log of colony forming units per gram of beef; error bars indicate the standard error of the mean.

In Figure 2 are shown the mean Log counts and the percentual bacterial distribution of *Pseudomonads*, *Enterobacteriaceae*, and lactic acid bacteria for each treatment over time. The mean log average and the bacterial composition found in the beef samples at packaging were similar among the four treatments. The initial microbial load of the samples was low, EB varied between 0.2 and 0.4 log CFU/g, PSE varied between 0.8 and 1.0 log CFU/g, and LAB varied between 0.1 and 0.7 log CFU/g. For all the treatments, after 28 days of storage at refrigerated conditions, a significant (*p* < 0.05) increase in the number of PSE, EB, and LAB was observed. The increase in PSE and EB in the VPAM group was significantly greater (*p* < 0.05) than the increase in the VP group, while the LAB number increase was not significantly different (*p* > 0.05) among treatments. At day 28 of storage, the proportion of *Pseudomonads* in the samples of the VP groups decreased, and the estimated bacterial distributions for VP 120R samples were 42% PSE, 57% LAB, and 1% EB, and for VP 28R+92F, they were 30% PSE, 67% LAB, and 3% EB. On the contrary, for the VPAM groups, there was an increase (*p* < 0.05) in the *Pseudomonads* proportion, accounting for 97% of the bacterial population.

In samples stored at −20 °C after day 28, both the amount and distribution of the bacterial population remained almost constant over time; only the LAB number in the VPAM 28R+92F treatment had a significant decay at day 120 (Table S1). Bacterial log counts at the end of the storage period were 2.3 ± 0.4 log CFU/g for EB, 2.6 ± 0.3 log CFU/g for PSE, and 2.2 ± 0.5 log CFU/g for LAB in the VP28R+92F group, as well as 2.9 ± 0.3 log CFU/g for EB, 5.2 ± 0.1 log CFU/g for PSE, and 1.1 ± 0.2 log CFU/g for LAB in the VPAM 28R+92F group. Only PSE log counts were significantly different (*p* < 0.05) between the treatments (Table S1).

In frozen samples after day 28 of storage, the three families of bacteria analyzed were able to grow. In VP 120R beef samples, EB evolved from 1.7 ± 0.2 log CFU/g at day 28 to 4.8 ± 0.9 log CFU/g at day 120; PSE evolved from 3.4 ± 0.1 log CFU/g to 5.4 ± 0.4 log CFU/g; and LAB evolved from 3.5 ± 0.6 to 5.3 ± 0.2 log CFU/g. In VPAM 120R beef samples, the number of EB evolved from 3.8 ± 0.2 log CFU/g at day 28 to 6.9 ± 0.9 log CFU/g at day 120; PSE evolved from 5.9 ± 0.1 log CFU/g to 7.1 ± 0.4 log CFU/g; and LAB evolved from 3.9 ± 0.6 to 5.6 ± 0.2 log CFU/g. Both PSE and EB bacterial log counts in VPAM samples were, at every sampling time, significantly greater (*p* > 0.05) than in VP samples. At the end of the storage period, the bacterial population of VP 120R samples was composed of 42% of LAB, 46% of PSE, and 12% of EB, while the bacterial population of VPAM 120R samples was 64% PSE, 34% EB, and only 2% of LAB.

### 3.2. Microbiome Richness and Evenness

**Figure 3:**
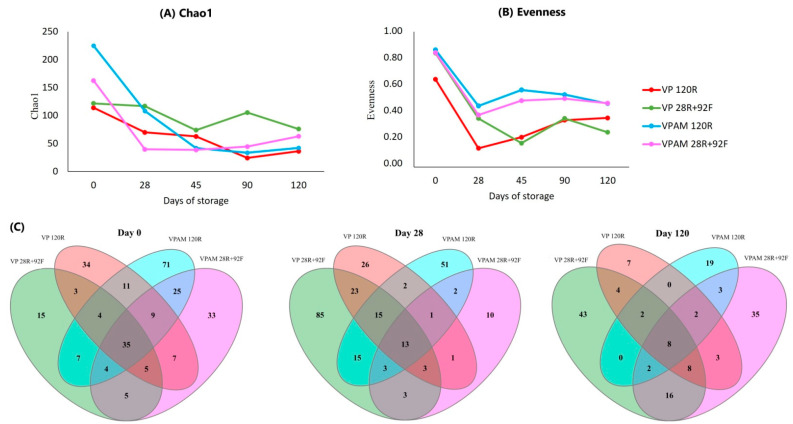
Chao 1 (**A**) and evenness (**B**) alpha diversity of the bacterial microbiome of meat samples at amplicon sequence variant level (ASV). Venn diagram (**C**) showing the unique and shared genera between treatments on day 0, 28, and 120 of storage. Treatments: vacuum packaging for 120 days (refrigerated) (VP 120R), vacuum packaging for 28 days (refrigerated) + 92 days (frozen) (VP 28R+92F), vacuum packaging with antimicrobial for 120 days (refrigerated) (VPAM 120R), vacuum packaging with antimicrobial for 28 days (refrigerated) + 92 days (frozen) (VPAM 28R+92F).

The evolution of Chao1 and evenness for all treatments at the ASV level is presented in Figure 3. Chao1 is an indicator of species richness (total number of species in a sample), and evenness refers to how equally abundant those species are in a sample. The microbiome Chao1 richness estimator was affected (*p* < 0.05) by packaging on days 0, 28, and 45 (Figure 3A). Between-treatment differences in microbiome richness on day 0 were attributed to the natural variation of the original samples. For treatments with VPAM meat samples, the number of ASVs was lower (*p* < 0.05) than those VPs on day 28 (VPAM = 74 ± 48; VP = 94 ± 33) and on day 45 (VPAM = 40 ± 2; VP = 68 ± 4). In addition, both groups of VP samples (120R and 28R+92F) had lower (*p* < 0.05) evenness than VPAM samples on day 28 (0.25 ± 0.28 and 0.48 ± 0.06, respectively) and on day 45 (0.20 ± 0.12 and 0.53 ± 0.07, respectively) (Figure 3B). The Venn diagram analysis on day 28 (Figure 3C) revealed that comparing VP and VPAM samples for each storage condition, there were 53 (VP vs. VPAM 120R) and 138 (VP vs. VPAM 28R+92F) genera observed only in VP samples. This result should be interpreted with caution since VP samples had already presented greater richness on day 0 before implementing the treatments.

Chao1 was different (*p* < 0.05) among treatments on day 90, where VP 28R+92F had greater ASV richness (106 ± 34) compared to the average of the other treatments (34 ± 10 and 15 ± 5, respectively). In addition, evenness tended (*p* = 0.07) to be reduced in VP samples. On the last day of sampling (day 120), storage had a significant impact on microbial richness (*p* < 0.05). Frozen meat samples showed greater (*p* < 0.05) Chao1 values compared to those refrigerated at ASV level (70 ± 9 and 31 ± 4, respectively). Paired comparison of refrigerated and frozen samples with each packaging condition in the Venn diagram on day 120 revealed that there were 61 (VP 28R+92F vs. 120R) and 62 (VPAM 28R+92F vs. 120R) genera observed in frozen samples that were not found in those refrigerated. On the other hand, only 11 (frozen) and 21 (refrigerated) genera were present in VPAM samples, but not found in those without antimicrobial (Table S2). 

### 3.3. Microbiome Composition

**Figure 4:**
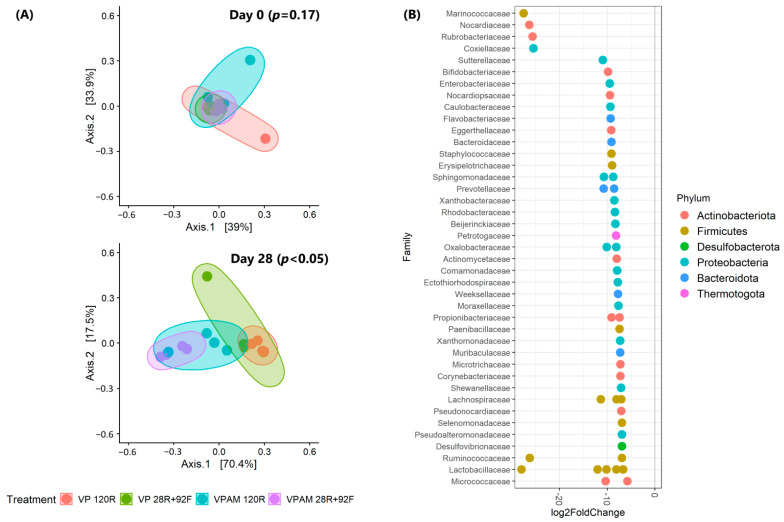

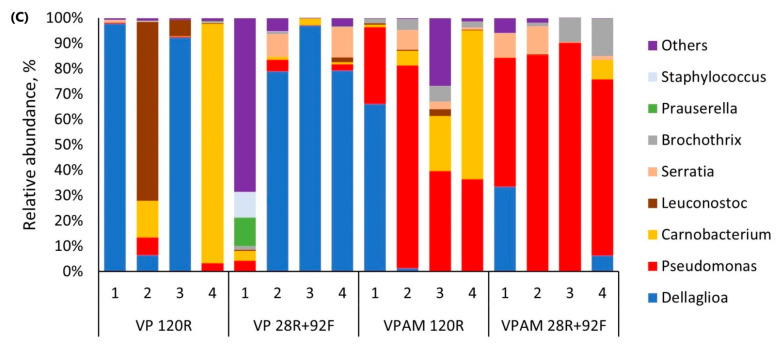
(**A**) The principal coordinate plot of beta diversity measured by weighted UniFrac distances at amplicon sequence variants (ASV) level on days 0 (top) and 28 (bottom). (**B**) Significant (*p* < 0.05) differential genera in meat vacuumed packaged with or without antimicrobial. Genera are grouped by family (y-axis) and color-coded according to the phyla they belong to. Negative log_2_fold values mean enrichments in samples without antimicrobial. (**C**) Relative abundance (%) of most predominant bacterial genera in individual meat samples for each treatment on day 28 of storage. Treatments: vacuum packaging for 120 days (refrigerated) (VP 120R), vacuum packaging for 28 days (refrigerated) + 92 days (frozen) (VP 28R+92F), vacuum packaging with antimicrobial for 120 days (refrigerated) (VPAM 120R), vacuum packaging with antimicrobial for 28 days (refrigerated) + 92 days (frozen) (VPAM 28R+92F).

On day 0, meat microbial beta diversity was not affected (*p* > 0.05) by treatment (Figure 4A, top). At the beginning of storage, a total of 267 genera were identified across all meat samples. The top-10 major bacteria included *Prauserella* (relative abundance: 13.9%), PSE (9.2%), *Staphylococcus* (8.9%), *Dellaglioa* (6.1%), *Lipingzhangella* (5.7%), *Pelomonas* (4.3%), *Alteribacillus* (4.6%), *Acinetobacter* (4.1%), *Prevotella* (3.1%), and *Corynebacterium* (2.5%), which combined accounted for 62.5% of the bacterial community (min. 42.7%; max. 96.9%). After 28 days of refrigerated storage for all samples, meat samples were clustered (*p* < 0.05) by packaging type (Figure 4A, bottom). An amount of 54 genera were different (*p* < 0.05) when comparing VP and VPAM samples (Figure 4B). All of them significantly reduced their abundance in VPAM samples. On the other hand, PSE tended to increase in samples packaged with antimicrobial (*p* = 0.08). *Dellaglioa* (56.5%) and PSE (60.3%) were recognized as the main genera for VP and VPAM, respectively (Figure 4C).

**Figure 5:**
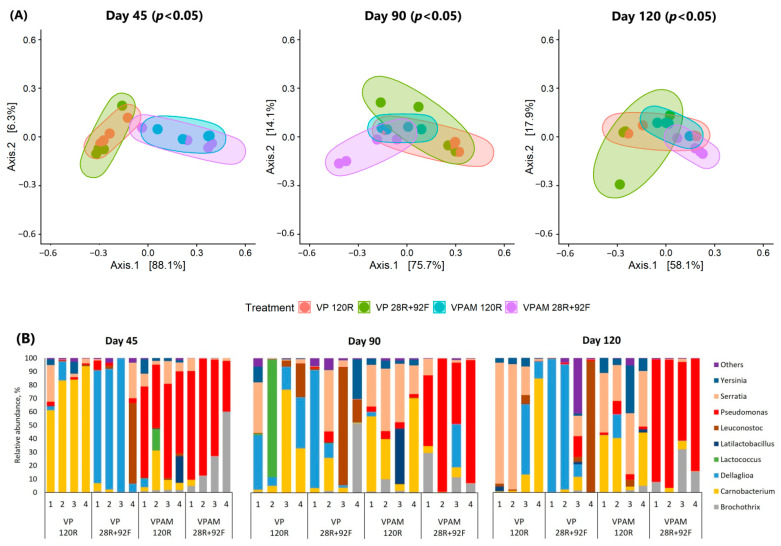
(**A**) The principal coordinate plot of beta diversity measured by weighted UniFrac distances at amplicon sequence variants (ASV) level on days 45, 90, and 120. (**B**) Relative abundance (%) of most predominant bacterial genera in individual meat samples for each treatment within each sampling time. Treatments: vacuum packaging for 120 days (refrigerated) (VP 120R), vacuum packaging for 28 days (refrigerated) + 92 days (frozen) (VP 28R+92F), vacuum packaging with antimicrobial for 120 days (refrigerated) (VPAM 120R), vacuum packaging with antimicrobial for 28 days (refrigerated) + 92 days (frozen) (VPAM 28R+92F).

Beta-diversity analysis revealed a significant clustering by treatment on days 45, 90, and 120 (Figure 5A). This was supported by 10, 7, and 17 genera that were impacted (*p* < 0.05) by treatment on those sampling days, respectively (Table S3), corresponding to the phyla *Proteobacteria* (n = 9 genera), *Firmicutes* (n = 7), *Bacteroides* (n = 5), and *Actinobacteriota* (n = 4). A small number of genera represented >90% of the common and dominant meat microbiomes in all treatments (Figure 5B). Statistical information of log_2_ fold changes for each genus based on pairwise comparisons between treatments is given in Table S4. *Carnobacterium* in VP 120R samples (relative abundance: 80.5 ± 13.9%) and *Dellaglioa* in VP 28R+92F samples (70.1 ± 42.9%) dominated (*p* < 0.05) the meat microbiome in packages without antimicrobial on day 45. On the other hand, PSE was more abundant (*p* < 0.05) in VPAM samples compared to those VP on day 45 (65.4 ± 16.5% and 2.3 ± 2.2%, respectively). Between VPAM treatments, *Brochothrix* was enriched (*p* < 0.05) in frozen samples (26.2 ± 24.5%) compared to those refrigerated (1.6 ± 0.5%).

On day 90, *Leuconostoc* (*p* < 0.05) was enriched in VP samples. In addition, PSE was more abundant (*p* < 0.05) in VPAM 28R+92F (72.1 ± 26.7%) than in the rest of the treatments (average 2.1 ± 2.0%). At the end of storage (day 120), VPAM 28E+92C samples were the most different compared to those in the rest of the treatments. This was supported by increased (*p* < 0.05) abundance of PSE and *Brochothrix* in VPAM 28E+92C compared to VP 28E+92C, VP 120E, and VPAM 120E, continuing the trend observed on days 45 and 90. Further, the abundance of *Yersinia* and *Serratia*, both EB, was higher (*p* < 0.05) in samples refrigerated at the end of storage compared to those frozen, regardless of the packaging type. 

### 3.4. Prediction of Metabolic Functions of Microbiomes at the End of Storage

**Figure 6:**
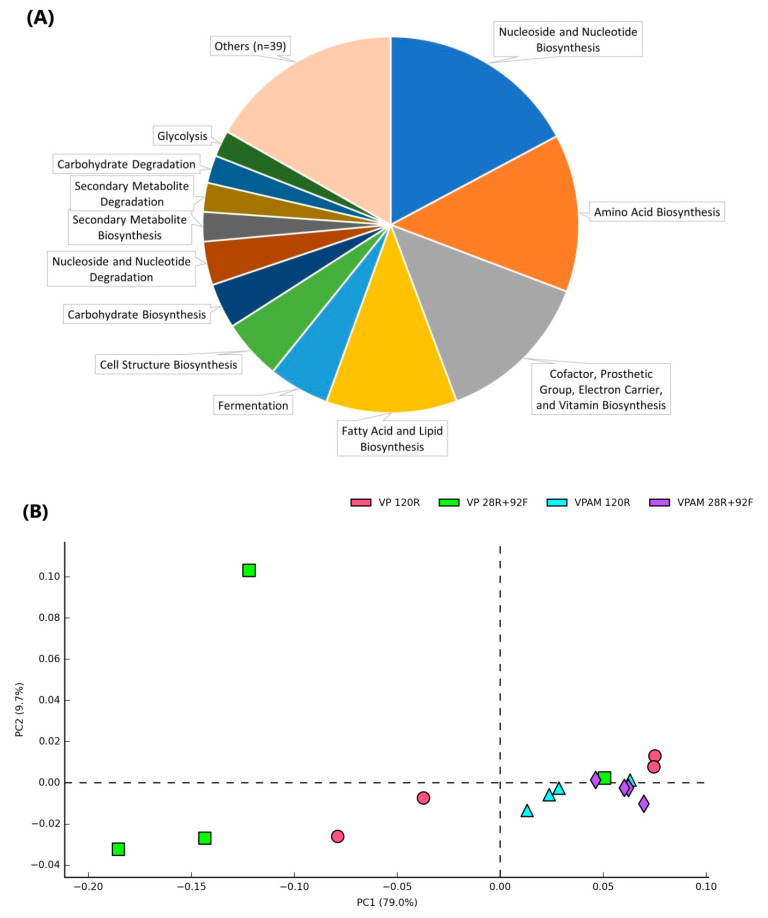

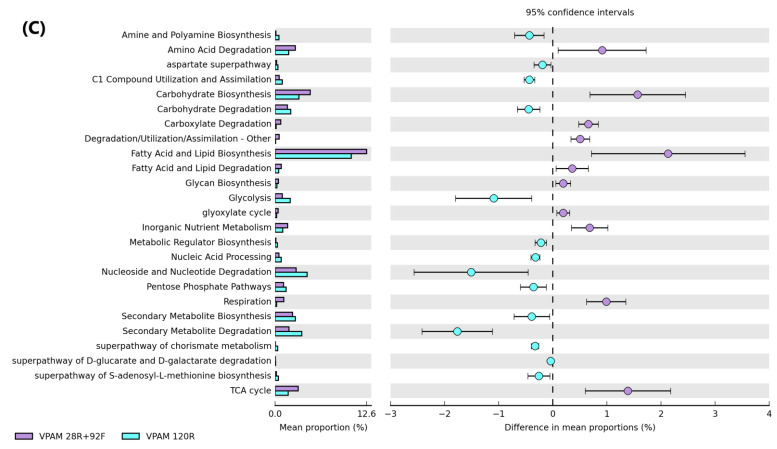
Predicted metabolic functions (level 2 KEGG pathways using PICRUSt2) based on the microbiome of meat samples. (**A**) Relative abundance across all samples (pathways with <2% relative abundances are included in “Others”), (**B**) Principal coordinate analysis of the predicted function of meat bacterial communities separated by treatment, (**C**) Mean abundance and difference of significantly affected pathways between VPAM 28R+92F and VPAM 120R samples. Treatments: vacuum packaging for 120 days (refrigerated) (VP 120R), vacuum packaging for 28 days (refrigerated) + 92 days (frozen) (VP 28R+92F), vacuum packaging with antimicrobial for 120 days (refrigerated) (VPAM 120R), vacuum packaging with antimicrobial for 28 days (refrigerated) + 92 days (frozen) (VPAM 28R+92F).

PICRUSt2 was used to determine the similarities of predicted metabolic functions of the meat microbial communities between treatments on day 120. A total of 51 level 2 KEGG pathways were identified across all samples dominated by biosynthesis of nucleosides and nucleotides (mean relative abundance ± s.d.: 17.2 ± 6.4%), amino acids (13.6 ± 2.3%), cofactors and vitamins (13.5 ± 4.6%), and fatty acid and lipids (11.2 ± 1.6%) (Figure 6A). Nucleoside and nucleotide (3.7 ± 1.4%) and secondary metabolites (2.5 ± 1.4%) were the main compounds associated with mechanisms of degradation. Treatment separation in the PCoA plot (Figure 6B) was driven by nine level 2 KEGG pathways significantly affected by treatment, including the categories of degradation (amino acids, secondary metabolites, carboxylate, and others), glycan biosynthesis, chorismate metabolism, fermentation, respiration, and nucleic acid processing. 

Pairwise comparisons between treatments showed that VPAM 28R+92F samples formed a distinct cluster and were separated from VPAM 120R samples (*p* < 0.05) driven by 20/51 (39%) level 2 KEGG pathways that were different (*p* < 0.05). Considering the most abundant functions that were significantly affected, pathways related to carbohydrate and lipid biosynthesis were enriched in VPAM 28R+92F samples, while pathways associated with degradation of nucleosides/nucleotides (purine and pyrimidine) and secondary metabolites (d-fructuronale, d-galactarate, d-galacturonate, and d-glucarate) were more abundant in VPAM 120R samples (Figure 6C). Additionally, four pathways associated with the generation of precursor metabolite and energy (respiration, TCA cycle, glycolysis, and pentose phosphate pathway) were different between VPAM 28R+92F and VPAM 120R samples. Respiration and TCA cycle were more abundant (*p* < 0.05) in VPAM 28R+92F samples. Glycolysis and pentose phosphate pathway, on the other hand, were enriched (*p* < 0.05) in VPAM 120R samples. Additionally, degradation of amino acids and lipids (beta oxidation and androstenedione degradation) tended (*p* = 0.06) to be greater in VPAM 28R+92F samples than in VPAM 120R, while the aspartate superpathway tended (*p* = 0.06) to be enriched in the latter. A closer examination of level 3 KEGG pathways within amino acid degradation showed that arginine, leucine, and tyrosine degradation pathways were enriched in VPAM 28R+92F samples, whereas degradation of arginine, ornithine, and putrescine were more abundant in VP 120R samples. 

## 4. Discussion

The improvement of the microbiological quality and extension of the shelf-life of VP beef meat is crucial to supply distant export markets with high-quality and safe meat. It has been previously shown that the growth of microorganisms and their metabolic activity can significantly affect the shelf life and quality of VP beef meat stored under refrigerated conditions [5,6,36]. In the present study, we investigated the effect of the storage temperature and the packaging type on the microbial community composition of Uruguayan VP beef stored for 120 days. Despite the long storage, the different treatments were able to maintain an appropriate visual appearance of the meat during the study. This outcome could be associated with the low microbial load on meat samples at the beginning of the study, which reflected good hygienic practices of the abattoir and the proper temperature control during storage. The farm practices, animal, slaughter environment, and abattoir workers are among the major sources of contamination that drive the initial microbiome in VP meat [8]. Then, the progression from an initial diverse microbial community composition to a meat microbiome primarily dominated by few genera agrees with results from other studies [7,9]. In our study, 268 unique genera were identified on day 0, whereas 153 were present on day 120. Moreover, seven genera dominated the meat microbiome at the end of storage regardless of treatment: *Pseudomonas*, *Serratia*, *Carnobacterium*, *Dellaglioa*, *Leuconostoc*, *Yersinia*, and *Brochothrix*. This groups of cold-tolerant bacteria able to survive, grow, and dominate the meat microbiome during storage have been defined as specific spoilage organisms and represent only a fraction of the initial microbiome [7]. 

The results of the microbial counts and amplicon sequences on day 28 showed that PSE was favored under refrigerated VPAM conditions. PSE has been frequently isolated from aerobically stored meat products [37] and tends to dominate spoilage-associated microbiomes under conditions supporting aerobic growth. In the present study, the VPAM film had a high oxygen transmission rate (OTR), suggesting the presence of oxygen within the package favoring the proliferation of PSE, which could not be prevented by the addition of the antimicrobial in VPAM packages. As a result, ASV richness as a proxy of species richness was reduced in VPAM samples due to the predominance of PSE during early storage. Moreover, the antimicrobial was effective in reducing the abundance of 54 genera. The antimicrobial mechanism was based on the release of silver ions that permeate through bacterial cell walls, deactivating essential energy-producing metabolic enzymes and preventing bacteria from growing [21,38]. Although we did not find an effect of the antimicrobial on PSE, [21] reported the effectiveness of the same antimicrobial in reducing total plate counts in chicken breast fillets. Upon onset of freezing on day 28, PSE continued dominating the microbiome in frozen VPAM samples, but its relative abundance was sequentially decreasing in those refrigerated as a result of EB growth, specially *Serratia* spp. and *Yersinia* spp. Overgrowth of EB in refrigerated samples is a concern not only from the microbiological shelf-life standpoint, but also as an indicator of meat safety [39].

The onset of freezing on day 28 inhibited microbial growth demonstrated by the plateau in LAB, EB, and PSE growth. Although freezing does not eliminate microbial organisms, it prevents microbial proliferation, thereby reducing the rate of spoilage compared to refrigerated meat [23,24,40]. As a result, the microbiome composition was relatively stable in frozen VP and VPAM samples when comparing days 28 and 120, dominated by LAB and PSE, respectively. It is well known that VP storage can favor the dominance of a facultative anaerobic population, mainly LABs, which outcompete other spoilage-related groups, such EB, PSE, and *Brochothrix* [10,12]. In a previous study [4], LAB increased from 1.15 log CFU/cm^2^ in VP beef primals to 5 log CFU/cm^2^ after 26 weeks of storage at −0.5 °C with no or slight visual discoloration evaluated by a sensory panel.

Overall, the combined results of the culture and sequencing approach showed that the spoilage-related microbial groups had different evolution depending on the packaging type. At the end of storage, PSE, *Serratia,* and *Brochothrix* were identified as more abundant in VPAM samples (film with higher OTR). On the other hand, LABs were dominant in beef stored under VP conditions with low OTR. By comparing the results obtained from the culture- and molecular-based approach, it can be concluded that amplicon sequencing identified a larger number of microbial genera, providing an ecological insight into spoilage-related bacteria in meat. However, the potential occurrence of sequences from dead cells in 16S rRNA analysis makes the cultivation approach complementary and necessary to quantify microbial growth [10]. The combination of both approaches showed that behind each CFU/gram value reported by cultivation for each indicator bacteria may be different microbial genera. Among LABs, *Dellaglioa* dominated VP 120R samples on day 28, while *Carnobacterium* and *Leuconostoc* were found among the dominant LABs during mid and late storage.

Although the population of EB, LAB, and *Pseudomonas* reached between 5 and 7 log CFU/g in refrigerated samples at the end of storage, no visible signs of deterioration were observed (i.e., slime, discoloration, or off-odors). These characteristics usually become evident when spoilage bacteria have exhausted the glucose present in meat and begin to utilize nitrogen compounds, such as amino acids [39]. Based on our results, the end of shelf-life of refrigerated samples was closer compared with those that were kept frozen from day 28 of storage, which presented lower bacterial counts at the end of storage. The curves fitted for PSE, LAB, and EB growth in Figure 1 for frozen samples indicate a stationary phase around 2-3 log CFU/g, except for PSE in VPAM 28R+92F (~5 log CFU/g), in contrast to the refrigerated samples that reached the stationary phase at levels of 5–7 log CFU/g for all indicator bacteria. Additionally, the microbiome of VP 28R+92F samples was dominated by LAB on day 120. While LABs are recognized as causative agents of meat spoilage, they also delay spoilage caused by other bacteria through the production of organic acids and bacteriocins [10,41,42].

Analysis of the predicted metabolic functions of the microbiomes using PICRUSt2 showed that VPAM 28R+92F and VPAM 120R samples clustered apart. This agrees with the different composition of the microbiomes on day 120, dominated by PSE and *Brochothrix* in VPAM 28R+92F, whereas a mixture of LAB and EB was found to be prevalent in VPAM 120R samples. This suggested that the temperature of storage had a greater impact than the type of packaging on shaping the function of the microbiomes. VP samples had greater within-group variation in microbiome composition and predicted metabolic activity. This was clear in VP 120R samples on day 120, where 50% of the samples were dominated by *Serratia,* and the other 50% were dominated by LAB. This could be attributed to random variation and/or the presence of oxygen in certain parts within VP packages associated with imperfect film adhesion or residual oxygen in the beef muscle [19,43].

Among the most abundant significantly affected pathways, those related to degradation were enriched under refrigerated conditions (i.e., secondary metabolite, nucleoside, and carbohydrate degradation), whereas biosynthesis pathways were enriched under freezing temperatures (i.e., lipid and carbohydrate biosynthesis). This could be related to the higher total counts of indicator bacteria in VPAM 120R as microorganisms slow their growth rate and reduce nutrient synthesis as the competition for nutrients increases (i.e., stationary phase of microbial growth) [44]. However, it is possible that some of the genera detected in this study by next-generation sequencing could have been from dead or metabolically inactive bacterial groups, as this approach does not allow for distinguishing dead or living bacteria or whether they are metabolically active or simply present (inactive) in the microbiome [45]. As a result, the predicted functions obtained in frozen samples on day 120 could be a ‘picture’ of the microbiome activity at the time of freezing onset (day 28).

## 5. Conclusions

This study allowed the characterization of the vacuum-packaging beef microbial spoilage during long-term storage using culture-based techniques and next-generation sequencing. All combinations of packaging and temperature resulted in microbiological levels below the traditional shelf-life limits after 120 days of storage. However, the combination of low-O_2_ permeability vacuum-packaging and refrigeration-then-frozen was the strategy that resulted in lower microbial counts at the end of the storage period, allowing one to extend the meat storage-life. On the other hand, the addition of the antimicrobial to inhibit microbial growth was not enough to compensate for the high-O_2_ permeability of the VPAM film, especially at refrigeration temperatures. Future research needs to focus more in depth on the relationship between frozen storage (with previous aging) and eating quality to determine if prolonged frozen storage could have any detrimental effect on the organoleptic characteristics of meat.

## Figures and Tables

**Table 1:** Carcass characteristics of the steers (N = 40).

Characteristics	Number of Steers
Dentition (number of teeth)	
2	26
4	14
Conformation ^a^	
A	40
Degree of finishing ^b^	
1	12
2	28

^a^ Conformation according to the Uruguayan grading system [27], I-N-A-C-U-R: from large muscle development to lack of muscle development. ^b^ Degree of finishing according to the Uruguayan grading system [27] from 0: lack of fat cover to 4: excessive finishing.

## Data Availability

Raw sequencing data (80 paired-read sequences) obtained from genomic DNA in the present study are publicly available on Jan-2024 or upon publication (whichever is first) at the National Center for Biotechnology Information (NCBI) database with Bio Project accession number PRJNA914268.

## Supplementary Materials

The following supporting information can be downloaded at: https://www.mdpi.com/article/10.3390/foods12040694/s1, Table S1: *Enterobacteriaceae* (EB) *Pseudomonads* sp (PSE) and Lactic Acid Bacteria (LAB) over time in beef treated with different packaging and refrigeration systems stored for 120 days; Table S2: Unique genera found in each treatment on day 120 of storage; Table S3: Log_2_ fold change in genera significantly affected by treatment on days 45, 90, and 120 of storage; Table S4: Log_2_ fold change (LFC) of paired comparison of top genera among different treatments for each sampling day.
