# Supplementary material for: Microbiological Changes during Long-Storage of Beef Meat under Different Temperature and Vacuum-Packaging Conditions
Source: Foods. 2023 Feb 6;12(4):694. doi: 10.3390/foods12040694 (PMC9955083; doi:10.3390/foods12040694)
Supplement: Supplementary file 1 [file foods-12-00694-s001.zip › supplemenary data/Table S1.pdf]

Table S1. *Enterobacteriaceae* (EB) *Pseudomonads* sp (PSE) and Lactic Acid Bacteria (LAB) over time in beef treated with different packaging and refrigeration systems stored for 120 days.

Enterobacteriaceae (Log CFU/g)					
	Treatments				
Sampling Day	VP 120R	VP 28R+92F	VPAM 120R	VPAM 28R+92F	*P*
0	0.38 ± 0.09 $^{a, y}$	0.16 ± 0.06 $^{a, y}$	0.22 ± 0.09 $^{a, w}$	0.22 ± 0.07 $^{a, y}$	*0.2814*
28	1.74 ± 0.22 $^{a, y}$	1.93 ± 0.44 $^{a, z}$	3.84 ± 0.22 $^{b, x}$	3.62 ± 0.27 $^{b, z}$	*0.0001*
45	3.57 ± 0.28 $^{b, z}$	1.74 ± 0.40 $^{a, y, z}$	5.45 ± 0.28 $^{c, y}$	2.90 ± 0.47 $^{a, b, z}$	*0.0001*
90	4.86 ± 0.34 $^{b, z}$	1.73 ± 0.45 $^{a, y, z}$	6.57 ± 0.34 $^{c, z}$	2.79 ± 0.44 $^{a, z}$	*0.0001*
120	4.76 ± 0.86 $^{b, z}$	2.31 ± 0.43 $^{a, z}$	6.87 ± 0.86 $^{c, z}$	2.89 ± 0.32 $^{a, b, z}$	*0.0001*
P	*0.0001*	*0.0064*	*0.0001*	*0.0001*	

Pseudomonads (Log CFU/g)					
	Treatments				
Sampling Day	VP 120R	VP 28R+92F	VPAM 120R	VPAM 28R+92F	*P*
0	0.96 ± 0.20 $^{a, x}$	0.92 ± 0.10 $^{a, y}$	0.83 ± 0.20 $^{a, w}$	0.83 ± 0.14 $^{a, y}$	*0.9198*
28	3.38 ± 0.08 $^{a, y}$	2.96 ± 0.11 $^{a, z}$	5.85 ± 0.08 $^{c, x}$	5.18 ± 0.04 $^{b, z}$	*0.0001*
45	3.75 ± 0.15 $^{b, y}$	2.26 ± 0.24 $^{a, z}$	6.29 ± 0.15 $^{d, x, y}$	5.44 ± 0.08 $^{c, z}$	*0.0001*
90	5.13 ± 0.22 $^{b, z}$	2.59 ± 0.23 $^{a, z}$	6.80 ± 0.22 $^{c, y, z}$	5.26 ± 0.08 $^{b, z}$	*0.0001*
120	5.35 ± 0.44 $^{b, z}$	2.56 ± 0.28 $^{a, z}$	7.14 ± 0.44 $^{c, z}$	5.23 ± 0.08 $^{b, z}$	*0.0001*
P	*0.0001*	*0.0001*	*0.0001*	*0.0001*	

Lactic Acid Bacteria (Log CFU/g)					
	Treatments				
Sampling Day	VP 120R	VP 28R+92F	VPAM 120R	VPAM 28R+92F	*P*
0	0.61 ± 0.06 $^{b, w}$	0.05 ± 0.04 $^{a, y}$	0.68 ± 0.06 $^{b, x}$	0.25 ± 0.08 $^{a, x}$	*0.0001*
28	3.52 ± 0.61 $^{a, x}$	3.30 ± 0.39 $^{a, z}$	3.85 ± 0.61 $^{a, y}$	3.02 ± 0.33 $^{a, z}$	*0.5573*
45	3.90 ± 0.44 $^{a, b, x, y}$	3.18 ± 0.54 $^{a, z}$	4.91 ± 0.44 $^{b, y, z}$	3.08 ± 0.09 $^{a, z}$	*0.0057*
90	6.16 ± 0.41 $^{b, y, z}$	2.72 ± 0.55 $^{a, z}$	5.45 ± 0.41 $^{b, z}$	3.02 ± 0.07 $^{a, z}$	*0.0001*
120	5.31 ± 0.21 $^{b, z}$	2.16 ± 0.49 $^{a, z}$	5.57 ± 0.21 $^{b, z}$	1.06 ± 0.20 $^{a, y}$	*0.0001*
P	*0.0001*	*0.0001*	*0.0001*	*0.0001*	

Numbers represent the mean ± standard error. $^{a, b, c, d}$ numbers within a row with different letters are significantly different (P<0.05). $^{w, x, y, z}$ numbers within a column with different letters are significantly different (P<0.05).
