# Supplementary material for: Microbiological Changes during Long-Storage of Beef Meat under Different Temperature and Vacuum-Packaging Conditions
Source: Foods. 2023 Feb 6;12(4):694. doi: 10.3390/foods12040694 (PMC9955083; doi:10.3390/foods12040694)
Supplement: Supplementary file 1 [file foods-12-00694-s001.zip › supplemenary data/Table S2.pdf]

Table S2. Unique genera found in each treatment on day 120 of storage

Vacuum packaged without antimicrobial		Vacuum packaged with antimicrobial	
**28 days refrigerated and 92 days frozen**	**120 days refrigerated**	**28 days refrigerated and 92 days frozen**	**120 days refrigerated**
Acholeplasma	Anaerostipes	[Eubacterium] brachy group	Agrilactobacillus
Achromobacter	Arthrobacter	[Ruminococcus] gnavus	Anaerophaga
Acinetobacter	Clostridium sensu stricto	[Ruminococcus] torques group	Armatimonas
Actinomyces	Desulfomicrobium	Acetobacter	BRH-c57
Aeromonas	Desulfovibrio	Acinetobacter	C. Methylopumilus
A-N-P-R*	Faecalibaculum	Actinomyces	CL500-29 marine
Alteribacillus	Flavobacterium	Aerococcus	Flexistipes
Ancylobacter	Janthinobacterium	Alistipes	Geotoga
Aquabacterium	Lachnospiraceae NC2004	Anaerostignum	Halanaerobium
Caloramator	Lachnospiraceae UCG-004	Anaerotruncus	Lactococcus
Candidatus Planktophila	Muribaculum	Anaplasma	Latilactobacillus
Caulobacter		Angelakisella	Limnobacter
Comamonas		Aquabacterium	Marinobacter
Corynebacterium		Arthrobacter	Marinobacterium
Curvibacter		Atopostipes	Modicisalibacter
Cutibacterium		Bifidobacterium	Mucinivorans
Deinococcus		Bilophila	Pantoea
Dermacoccus		Bradyrhizobium	Pseudarcicella
Dialister		Brevundimonas	Rahnella1
Empedobacter		Breznakia	Roseovarius
Enhydrobacter		Candidatus Soleaferrea	Sediminibacterium
Enterobacter		Clostridium sensu stricto 11	
Escherichia-Shigella		Comamonas	
Fusobacterium		Cutibacterium	
Glutamicibacter		Desulfovibrio	
Hafnia-Obesumbacterium		DEV114	
Herbaspirillum		dgA-11 gut group	
hgcI clade		Enterobacter	
Lachnospiraceae NK4A136		Erysipelothrix	
Lachnospiraceae UCG-008		Escherichia-Shigella	
Lawsonella		Faecalibacterium	
Lelliottia		Fusicatenibacter	
Lipingzhangella		Gardnerella	
Methylobacter		Gemella	
Methylobacterium		Glutamicibacter	
Methylotenera		Hafnia-Obesumbacterium	
Micrococcus		Harryflintia	
Mycoplasma		HT002	
Nissabacter		Janthinobacterium	
Olsenella		Lachnoclostridium	
Parabacteroides		Lachnospiraceae NK4A136	
Parasutterella		Lachnospiraceae UCG-009	
Pelomonas		Lactobacillus	
Porphyromonas		Lelliottia	
Prauserella		Limosilactobacillus	
Proteiniphilum		Natranaerovirga	
Pseudorhodobacter		Neisseria	
Psychrobacter		Nonlabens	
Rhodopseudomonas		Odoribacter	
Romboutsia		Owenweeksia	
Rubrobacter		Paludicola	
Saccharopolyspora		Parabacteroides	
Shewanella		Psychrobacter	
Soehngenia		Rothia	
Solitalea		Solitalea	
Sphingobium		Staphylococcus	
Staphylococcus		Stenotrophomonas	
Stenotrophomonas		Streptococcus	
Trichlorobacter		Tenacibaculum	
Trichococcus		Terrimicrobium	
Veillonella		UCG-008	
		Zymophilus	

* A-N-P-R: Allorhizobium-Neorhizobium-Pararhizobium-Rhizobium1
