# Supplementary material for: Microbiological Changes during Long-Storage of Beef Meat under Different Temperature and Vacuum-Packaging Conditions
Source: Foods. 2023 Feb 6;12(4):694. doi: 10.3390/foods12040694 (PMC9955083; doi:10.3390/foods12040694)
Supplement: Supplementary file 1 [file foods-12-00694-s001.zip › supplemenary data/Table S3.pdf]

Table S3. Log_2 fold change (±standard deviation) of genera significantly affected by treatment on days 45, 90, and 120 of storage.

Phylum	Order	Genus	Sampling time		
			Day 45	Day 90	Day 120
Actinobacteriota	*Frankiales*	*hgcI clade*	-	-	23.4±4.2
	Micrococcales	*Kocuria*	20.5±4.2	-	-
	Pseudonocardiales	*Prauserella*	23.1±4.2	16.5±4.3	-11.0±4.3
	Streptosporangiales	*Lipingzhangella*	23.1±4.2	-	-
Bacteroidota	*Bacteroidales*	*Bacteroides*	-	-	8.1±2.9
		Muribaculum	-21.5±4.2	-	-
		Prevotellaceae UCG-001	-	-	-19,2±4.2
	Lactobacillales	*Latilactobacillus*	-	-	-27.9±2.8
	Sphingobacteriales	*Solitalea*	-	-	23.6±4.2
Firmicutes	*Bacteroidales*	*Alloprevotella*	-	-13.6±4.3	19.1±4.2
	Lactobacillales	*Brochothrix*	10,2±1.6	7.8±1.7	10.4±1.6
		Carnobacterium	-8.7±2.1	-	-5.1±1.3
		Lactococcus	-	-8.5±2.2	-
		Leuconostoc	-	-7.7±1.8	-
	Pseudonocardiales	*Dellaglioa*	-	-	-5.8±2.1
	Staphylococcales	*Staphylococcus*	-	-	20.9±4.2
Proteobacteria	*Burkholderiales*	*Janthinobacterium*	-22.2±3.8	-	-
		Pelomonas	21.1±4.2	-	-
	Enterobacterales	*Enterobacter*	21.5±4.2	-	-
		Lelliottia	-	-	27.5±3.0
		Serratia	-	-	-5.7±1.3
		Yersinia	-	-	-5.2±1.8
	Pseudomonadales	*Acinetobacter*	-	-	7.6±2.2
		Pseudomonas	7.9±1.6	15.1±1.3	13.4±1.4
		Psychrobacter	-	19.9±4.2	24.5±4.2
