# Supplementary material for: Microbiological Changes during Long-Storage of Beef Meat under Different Temperature and Vacuum-Packaging Conditions
Source: Foods. 2023 Feb 6;12(4):694. doi: 10.3390/foods12040694 (PMC9955083; doi:10.3390/foods12040694)
Supplement: Supplementary file 1 [file foods-12-00694-s001.zip › supplemenary data/Table S4.pdf]

Table S4. Log_2 fold change (LFC)[1] of paired comparison of top genera among different treatments[2] for each sampling day.

		**Paired comparison**					
		**VP 28R+92F vs.**			**VPAM 28R+92F vs.**		**VP 120R vs.**
**Genus**	**Day**	**VP 120R**	**VPAM 28R + 92F**	**VPAM 120R**	**VP 120R**	**VPAM 120R**	**VPAM 120R**
Brochothrix	45	7.1±1.6	-	-	10.2±1.6	6.7±1.6	-
	90	-	-	-	7.8±1.7	5.9±1.7	-
	120	-	-7.9±1.6	-	10.4±1.6	9.2±1.6	-
Carnobacterium	45	-	6.7±2.1	-	-8.7±2.1	-	-
	90	-	-	-	-	-	-
	120	-	-	-	-	-	-
Dellaglioa	45	7.8±2.6	15.1±2.6	13.3±2.6	-	-	-
	90	-	-	-	-	-	-
	120	-	-	-	-	-	-
Leuconostoc	45	-	-	-	-	-	-
	90	-	10.5±1.8	9.4±1.8	-7.7±1.8	-	6.6±1.7
	120	-	-	-	-	-	-
Pseudomonas	45	5.0±1.6	-	-	7.9±1.6	-	-
	90	6.9±1.3	-8.2±1.3	-	15.1±1.3	10.4±1.3	-4.7±1.3
	120	5.3±1.4	-8.0±1.4	-	13.4±1.4	10.6±1.4	-
Serratia	45	-	-	-	-	-	-
	90	-	-	-	-	-	-
	120	-5.0±1.3	-	-	-5.7±1.3	-	-
Yersinia	45	-7.0±2.7	-	-6.3±2.7	-	-	-
	90	-	-	-	-	-	-
	120		-	-	-5.1±1.8	-5.2±1.8	

[1]LFC±SE: Log_2 Fold Change ± Standard Error. Positive LFC values mean greater abundance in the first term of the paired comparison, while negative LFC values mean greater abundance in the second term of the paired comparison.

[2]Treatments: vacuum packaging 120 days refrigerated (VP 120R), vacuum packaging 28 days refrigerated + 92 days frozen (VP 28R+92F), vacuum packaging with antimicrobial 120 days refrigerated (VPAM 120R), vacuum packaging with antimicrobial 28 days refrigerated + 92 days frozen (VPAM 28R+92F).
